# Effects of Feeding 5-Aminolevulinic Acid on Iron Status in Weaned Rats from the Female Rats during Gestation and Lactation

**DOI:** 10.3390/ani12202869

**Published:** 2022-10-21

**Authors:** Junhui Li, Yuhuai Xie, Min Li, Shaotao Zhang, Qun Cheng, Weiren Yang

**Affiliations:** 1 Shandong Provincial Key Laboratory of Animal Biotechnology and Disease Control and Prevention, College of Animal Science and Veterinary Medicine, Shandong Agricultural University, Daizong Street 61#, Tai’an 271018, China; 2 Department of Immunology, School of Basic Medical Sciences, Fudan University, Shanghai 200032, China; 3 College of Animal Science and Technology, Qingdao Agricultural University, Qingdao 266109, China

**Keywords:** 5-aminolevulinic acid, iron status, weaned rats, female rats

## Abstract

**Simple Summary:**

In order to develop novel iron supplementation methods for piglets, this study employed female Sprague–Dawley (SD) rats as a model to investigate the effects of 5-aminolevulinic acid (5-ALA) on iron status in weaned rats. The results indicated that fed 5-ALA diets to female rats could improve the blood parameters of weaned rats, increase the concentration of Hepcidin in the liver and serum, and promote the expression of iron-related genes in the liver, suggesting that 5-ALA may be an excellent functional additive to improve the iron status of animals. Nevertheless, these effects still need to be further validated in piglets.

**Abstract:**

Using female Sprague–Dawley (SD) rats as a model, the current study aimed to investigate whether feeding 5-aminolevulinic acid (5-ALA) to female SD rats during gestation and lactation can affect the iron status of weaned rats and provide new ideas for the iron supplementation of piglets. A total of 27 pregnant SD rats were randomly assigned to three treatments in nine replicates, with one rat per litter. Dietary treatments were basal diet (CON), CON + 50 mg/kg 5-ALA (5-ALA50), and CON + 100 mg/kg 5-ALA (5-ALA100). After parturition, ten pups in each litter (a total of 270) were selected for continued feeding by their corresponding mother, and the pregnant rats were fed diets containing 5-ALA (0, 50 and 100 mg/kg diet) until the newborn pups were weaned at 21 days. The results showed that the number of red blood cells (RBCs) in weaned rats in the 5-ALA100 group was significantly higher (*p* < 0.05) than that in the CON or 5-ALA50 group. The diet with 5-ALA significantly increased (*p* < 0.05) the hemoglobin (HGB) concentration, hematocrit (HCT) level, serum iron (SI) content, and transferrin saturation (TSAT) level in the blood of weaned rats, as well as the concentration of Hepcidin in the liver and serum of weaned rats and the expression of *Hepcidin* mRNA in the liver of weaned rats, with the 5-ALA100 group having the highest (*p* < 0.05) HGB concentration in the weaned rats, and the 5-ALA50 group having the highest (*p* < 0.05) Hepcidin concentration in serum and in the expression of *Hepcidin* mRNA in the liver of weaned rats. The other indicators between the 5-ALA groups had no effects. However, the level of total iron binding capacity (TIBC) was significantly decreased (*p* < 0.05) in the 5-ALA50 group. Moreover, the iron content in the liver of weaned rats fed with 5-ALA showed an upward trend (*p* = 0.085). In addition, feeding a 5-ALA-supplemented diet could also significantly reduce (*p* < 0.05) the expression of *TfR1* mRNA in the liver of weaning rats (*p* < 0.05), and the expression of *Tfr1* was not affected between 5-ALA groups. In conclusion, dietary supplementation with 5-ALA could improve the blood parameters, increase the concentration of Hepcidin in the liver and serum, and affect the expression of iron-related genes in the liver of weaned rats. Moreover, it is appropriate to add 50 mg/kg 5-ALA to the diet under this condition.

## 1. Introduction

Iron is one of the most abundant elements on earth and plays an irreplaceable role in maintaining normal cell function in animals. However, due to the faster growth of newborn piglets in the early stage, the iron storage capacity of piglets is insufficient to maintain their typical requirements, which will make the piglets susceptible to iron deficiency anemia [1,2]. A lack of iron in piglets will cause abnormal hematopoietic function, growth retardation, and low immunity, which will seriously affect the health of piglets [3,4]. This symptom is solved in the actual production process by intramuscular iron-dextran injection to the piglet [5]. However, this method has disadvantages. It is common for multiple pigs to share a needle during the injection process, which increases the risk of iatrogenic disease transmission. Moreover, improper injection can also cause a stress response or even death to the piglets [6]. Therefore, finding a new iron supplementation method or an enhancer that promotes iron absorption to replace piglet intramuscular iron injection has become a hot topic in the pig industry. 

5-Aminolevulinic acid (5-ALA) is a type of pure natural δ-amino acid widely existing in the biosphere. It is formed by the condensation of Succinyl-CoA and Glycine, which plays a significant role in heme’s biosynthesis [7]. The process of heme synthesis is restricted by 5-ALA synthetase. Based on the proposed mechanism, when 5-ALA synthase is limited, the addition of exogenous 5-ALA may provide an adequate amount of precursor to induce the synthesis of additional heme. Recent studies have shown that 5-ALA has many physiological functions, such as enhancing the activity of cytochrome P450 in the body, participating in the immune response of the body, and improving the bacterial community structure and the iron status in animals [8,9,10,11]. Studies on the improvement of iron status in animals have shown that the main effects of 5-ALA include improving physiological blood indexes and iron content in tissues. Wang’s research showed that addition of 90 mg/kg 5-ALA to pregnant sows’ diets significantly increased the hemoglobin (HGB) concentration and plasma iron content of piglets throughout the nursing phase [12]. Moreover, laying hens’ serum iron (SI) content increased quadratically after being placed on the diet with 5 mg/kg 5-ALA [13]. 

Therefore, the objectives of the current study were to use Sprague–Dawley (SD) rats as a model to explore whether feeding 5-ALA to female rats during gestation and lactation can improve the physiological blood indicators, serum and liver iron content, and liver iron metabolism-related gene expression in weaned rats. Through this study, we hope to further improve our understanding of the possible mechanism of 5-ALA to affect the body’s iron status and provide a theoretical basis for applying 5-ALA in pig production.

## 2. Materials and Methods

### 2.1. Animal, Dietary Treatment and Experimental Design

The experimental procedures used in this research were reviewed and approved by the Institutional Animal Care and Use Committee of Shandong Agricultural University (Identification code: # SDAUA-2020-0710, Date of approval: 10 July 2020).

A total of 27 pregnant SD rats were randomly assigned to three treatments groups in nine replicates with one rat per litter. Dietary treatments were basal diet (CON), CON + 50 mg/kg 5-ALA (5-ALA50), and CON + 100 mg/kg 5-ALA (5-ALA100). In this paper, the dose of 5-ALA is determined according to Chang et al. [10] and Wang et al. [12]. After parturition, ten pups from each litter (a total of 270) were selected for ongoing feeding and observation by their respective mother, and the pregnant rats were fed diets containing 5-ALA (0, 50 and 100 mg/kg diet) until the newborn pups were weaned at 21 days. All pregnant rats were housed individually and were free to eat and drink during feeding. The temperature was controlled at about 24 °C, and the relative humidity was about 55%. As shown in Table 1, the experimental diet formula was formulated according to Liu et al. [14]. After birth, the baby rats and their mothers were fed together in cages. The water and bedding material were changed every three days until the end of the experiment.

### 2.2. Determination of Blood Physiological Parameter

At the end of the experiment, two weaned rats from each repetition were chosen for blood collection from the canthus of eye, and the rats were anesthetized with isoflurane inhalation first. The blood samples were transferred to the vacuum tubes (Becton Dickinson, Franklin Lakes, NJ, USA) treated with ethylenediaminetetraacetic acid dipotassium anticoagulant (EDTAK_2_) and stored at 4 °C. The blood samples were gently shaken, and the number of red blood cells (RBCs), hemoglobin (HGB) concentration, and hematocrit (HCT) level were determined by an automatic hematology analyzer (Sysmex KX-21, Sysmex Corporation, Kobe, Japan).

### 2.3. Determination of Serum Iron Index and Liver Iron Content

At the end of the experiment, blood was collected from weaned rat and transferred to a non-heparinized tube and stored at room temperature to promote blood coagulation. The blood was centrifuged (D-37520, Kendro, Hanau, Germany) at 3000 r/min for 10 min to collect the serum, which was stored at −20 °C until assay. SI content and total iron-binding capacity (TIBC) level were measured using colorimetry using assay kits (Nanjing Jiancheng Bioengineering Institute, Nanjing, China).

TSAT(%) = (SI/TIBC) × 100% (g)/Carcass weight (kg) × 100%(1)

At the end of the experiment, immediately after the weaned rats were euthanized, the livers were removed and rinsed with ice-cold saline and stored at −20 °C for further analysis. According to AOAC, liver samples were dried at 65 °C and ground using a mortar and pestle [15]. As described previously by Armstrong, liver samples were digested by a microwave digestion system (Model MDS-81D; CEM Corp., Matthews, NC, USA) [16]. Ultra-pure water was used as a blank, and the iron standard solution was used as the reference standard. The iron concentration in the livers was measured using atomic absorption spectrophotometry (AAS Z-5000, Hitachi, Tokyo, Japan).

### 2.4. Determination of Hepcidin Content in Serum and Liver

After fixation in 4% paraformaldehyde (P1110, Solarbio, Beijing, China), the liver tissues were rinsed slowly with running water and successively went through 70%, 80%, 85%, 90%, 95%, and 100% alcohol to be dehydrated for several hours. After transparentization with xylene, the livers were embedded in paraffin and cut into slices of 5 mm. Perl’s iron staining was performed using the Prussian blue stain with diaminobenzidine (DAB) to localize iron in the livers, and a photomicrograph system (Motic Images Software, Motic China Group Co., Ltd., Xiamen, China) was used for the observation.

### 2.5. Immunohistochemistry

After fixation with 4% paraformaldehyde, the liver tissue sections were heated with 0.01 mol/L sodium citrate buffer (pH 6) for antigen retrieval for 20 min in a microwave oven. Tissue sections were blocked with 10% normal goat serum (zsgb-bio, Beijing, China) for non-specific binding. To block endogenous peroxidase activity, sections were incubated in 10% hydrogen peroxide (H_2_O_2_) for 1.5 h. The antibody Hepcidin (1:100, bs-8870R, BIOSS, Beijing, China) was dripped onto tissue sections and incubated overnight at 4 °C. Immunohistochemical analysis for samples was performed according to the tissue staining SP Kit instructions (spn-9001, zsgb-bio, Beijing, China); detection and immunolocalization of Hepcidin was performed using a polink-2 plus polymer horseradish peroxidase (HRP) rabbit system (pv-9002, zsgb-bio, Beijing, China). The sections were soaked using diaminobenzidine tetrachloride for DAB staining (pa110, Tiangen, Beijing, China). Finally, all sections were dehydrated and sealed with neutral gum and then visualized under a microscope.

### 2.6. Total RNA Extraction, cDNA Preparation, and Quantitative Real-Time Reverse Transcription–Polymerase Chain Reaction (qRT-PCR)

Liver samples were collected and stored at −80 °C. The frozen sample was cut into approximately 50 mg and placed in a centrifuge tube without RNA enzyme. Total RNA was extracted using a Trizol Kit (R401-01, Vazyme, Nanjing, China). The absorbance ratio at 260/280 nm was applied to assess the purity of RNA. The quality of total RNA was assayed by 1% agarose gel electrophoresis. All the primers were designed and synthesized by Sangon Biotech (Shanghai, China), and the *β-actin* gene was used as an internal reference. Primer sequences and lengths for amplifications are depicted in Table 2. cDNA synthesis was performed according to the instructions of a HiScript II Q RT SuperMix for the qPCR kit (R223-01, Vazyme, Nanjing, China). The qPCR reaction system (20 µL) was prepared according to the instructions of an SYBR^®^Premix Pro Taq Hs qPCR Kit (AG11701, Accurate, Changsha, China). Real-time fluorescent quantitative PCR was performed using a LightCycler^®^ 96 quantitative PCR machine (Roche, Switzerland) and amplification under the following conditions: initial denaturation at 95 °C for 30 s, 40 cycles of 95 °C, 5 s, and 60 °C 30 s. The mRNA-relative quantification amounts were expressed and calculated by 2^−∆∆CT^ [17]. 

### 2.7. Data Calculations and Statistical Analysis

Data were analyzed with a one-way ANOVA using SAS 9.2 statistical software (SAS Inst. Inc., Cary, NC, USA), and the significance of differences among groups was tested using Duncan’s multiple range tests. Statistically, *p* < 0.05 was accepted as significant, and tendency was discussed at 0.05 ≤ *p* ≤ 0.10. All data within the different groups were reported as mean, standard error of the mean (mean, SEM).

## 3. Results

### 3.1. Blood Physiological Parameters of Weaned Rats

As shown in Table 3, the number of RBCs in the weaned rats of the 5-ALA100 group was significantly higher (*p* < 0.05) than that in the CON or 5-ALA50 group. In addition, 5-ALA-supplemented diets significantly increased (*p* < 0.05) the HGB concentration and HCT level in the blood of weaned rats, with the highest (*p* < 0.05) HGB concentration occurring in the 5-ALA100 group. However, there was no significant (*p* > 0.05) difference between the 5-ALA50 and 5-ALA100 groups at the HCT level.

### 3.2. Liver and Serum Iron-Related Indicators of Weaned Rats

**Figure 1:**
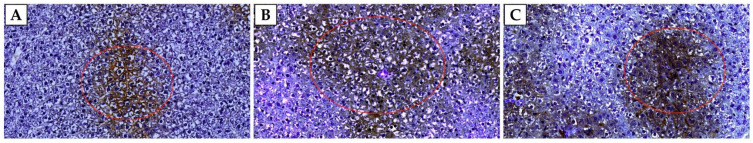
The content of iron in the liver of weaned rats. Iron was identified in the liver using Prussian blue staining with diaminobenzidine (DAB) enhancement (400×). (**A**), (**B**), and 100 (**C**) represent the sections from the rat fed with the basal diet with an addition of 0, 50, and 100 mg/kg 5-ALA, respectively. Stainable iron was circled.

As shown in Table 4, the SI content and TSAT level in the serum of weaned rats fed 5-ALA were significantly higher (*p* < 0.05) than the CON group; however, there is no significant (*p* > 0.05) impact on these indicators between 5-ALA50 and 5-ALA100, and the 5-ALA50 treatment significantly reduced the TIBC level (*p* < 0.05). Moreover, the iron content in the liver of weaned rats fed with 5-ALA has an upward trend (*p* = 0.085). As shown in Figure 1, after staining with the Prussian blue with DAB enhancement, the iron-containing parts appear brown under the microscope. Most of the stainable iron was located predominantly in the hepatocytes of weaned rats. The iron contents in the 5-ALA50 group were prominently higher than those in the CON and 5-ALA100 groups.

### 3.3. The Content of Hepcidin in the Liver and Serum of Weaned Rats

**Figure 2:**
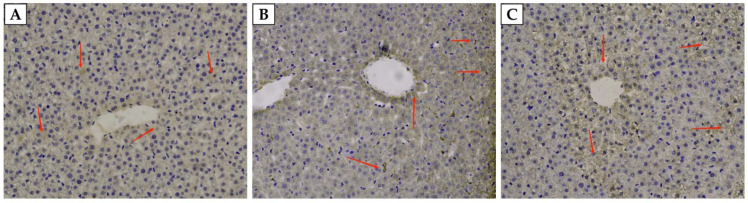
The content of Hepcidin in the liver of weaned rats (200×). (**A**), (**B**), and 100 (**C**) represent the sections from the rat fed with the basal diet with an addition of 0, 50, and 100 mg/kg 5-ALA, respectively. Arrow indicates Hepcidin-immunoreactive substances.

As shown in Table 5, the concentration of Hepcidin in the liver and serum of weaned rats fed with 5-ALA was significantly improved (*p* < 0.05) compared with the CON group, with the highest (*p* < 0.05) Hepcidin concentration of serum occurring in the 5-ALA50 group. However, there was no significant (*p* > 0.05) difference between the 5-ALA50 and 5-ALA100 groups’ Hepcidin concentration of the liver. As shown in Figure 2, our observations revealed that Hepcidin immunoreactive substances were mainly detected in the hepatocytes of weaned rats. The most notably chromogenic reaction of Hepcidin occurred in the 5-ALA50 group.

### 3.4. The Expression of Iron-Related Genes in the Liver of Weaned Rats

**Figure 3:**
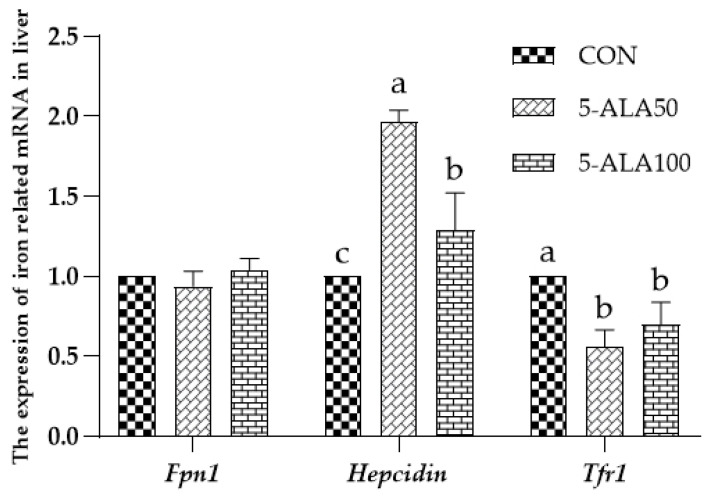
Effects of ALA on the expression of iron-related mRNA in the liver of weaned rats. Bars labeled with different letters represent a significant difference at *p* < 0.05. Dietary treatments were as follows: CON: basal diet; 5-ALA50: basal diet + 50 mg/kg 5-ALA; 5-ALA100: basal diet + 100 mg/kg 5-ALA.

As shown in Figure 3, no difference (*p* > 0.05) was observed concerning the expression of *Fpn1* mRNA in the liver among the treatment groups, whereas the expression of *Hepcidin* mRNA was significantly higher (*p* < 0.05) in the liver of 5-ALA-treated weaned rats than that of the CON group, and the 5-ALA50 treatment group had the highest (*p* < 0.05) *Hepcidin* gene expression. In addition, the treatment with 5-ALA could significantly reduce (*p* < 0.05) the expression of *Tfr1* mRNA in the livers of weaned rats. However, there was no significant (*p* > 0.05) difference between the 5-ALA50 and 5-ALA100 groups in the expression of *Tfr1* mRNA. 

## 4. Discussion

The physiological blood indexes were key indexes used to reflect the balance of the animals’ internal environment and the nutritional metabolism of the body. The blood is connected to the various tissues and organs of the whole body through the circulatory system and participates in various physiological activities such as respiration, nutrient and oxygen transport, defense against virus invasion, and the regulation of acid-base balance. Furthermore, hematological indices such as the number of RBCs, HGB concentration, and HCT level in the blood were also associated with anemia and iron metabolism in the body [18,19]. Blood indicators can mainly reflect iron status because iron is an essential element in hemoglobin biosynthesis and because approximately 73% of body iron is found in HGB [20]. Moreover, iron supplies can also directly affect erythrocyte synthesis. Consequently, a reduced erythrocyte production and a corresponding decrease in hematocrit will occur when iron is limited [21,22]. Previous studies have revealed that the cells’ treatment with 5-ALA could boost cellular endogenous heme biosynthesis [23]. It is well known that HGB is made up of four protein chains and four heme groups. Therefore, HGB may have an impact on blood indicators. Mateo et al. found that nursery pigs fed on 0.05% 5-ALA diets had a significantly higher number of RBCs than those fed on the basal diet, and there were no differences in HGB concentration [24]. Weaned piglets fed with 3 mg/kg ALA presented a significantly increased number of RBCs at d35, according to Yan and Kim’s report [25]. Weaned piglets fed with 0.2% 5-ALA had a significantly higher number of RBCs, HGB concentration, and HCT level than those in the control group, according to Min’s research [11]. It has also been shown that the level of RBC and HGB concentration increased linearly by increasing the 5-ALA supplementation levels in weaning piglets, and the number of RBCs and HGB concentration were higher in the 10 mg/kg 5-ALA group at d21 [26]. The present results show that providing rats with dietary 5-ALA supplementation can help them with better physiological blood parameters and that, according to the data from this study, the number of RBCs in weaned rat blood is unaffected by the 50 mg/kg 5-ALA diet. However, adding 5-ALA significantly improved other blood indicators such as the number of RBCs, HGB concentration, and HCT level in this experimental study. These results again verified that 5-ALA has an important role in blood, which may potentially influence the iron metabolism in body. The fact that 5-ALA serves as the universal precursor of heme metabolites may explain the mechanism [27]. However, heme is also an essential component of hemoglobin and has a catalytic and regulatory effect on cells, which plays a central role in terminal erythropoiesis [28,29]. When the 5-ALA synthesis is limited, the externally ingested 5-ALA can provide a sufficient precursor to induce the synthesis of additional heme, which affects the animals’ blood metabolism [30]. 

Iron is an essential factor for playing diverse functional roles in heme proteins and non-heme proteins, and the iron can also convert between ferrous iron (Fe^2+^) and ferric iron (Fe^3+^) in organisms [31]. It is also the main component crucial for hemoglobin and for the components of some enzymes that are also involved in the immune system’s regulation [32,33,34]. For iron homeostasis, the liver is the main organ, and its condition is closely related to how iron is distributed within the body [35]. The results of the present study showed that dietary 5-ALA supplementation had no significant effects on iron storage in the liver; however, there was a tendency to increase the iron content. After looking through the literature, there were very few studies on the effects of 5-ALA on the iron contents of animal tissues, and most of them focused on the effects on plasma iron content. Andersson et al. thought that iron is stored mainly in the bone marrow and liver, which can be estimated indirectly by using blood or serum [36]. Wang et al. found that the 90 mg/kg 5-ALA supplementation in sows’ diets increased the plasma iron contents at lactation d21 [12]. Another study showed that the plasma iron contents in weanling pigs were also increased by 5-ALA diets, with the 10 mg/kg 5-ALA treatment showing the highest concentration [26]. Extracellular iron enters the serum, converted by ceruloplasmin into ferric iron, which can bind with transferrin, being transported, and reaching tissues [37]. Similarly, a previous study also demonstrated that the dietary inclusion of 5-ALA could improve iron contents in the serum, liver, and breast meat of broilers [38]. SI or TIBC and other indicators are the common biomarkers for assessing whether the body is iron deficient or not, and these biomarkers may fluctuate with an acute or chronic infection or inflammation [39]. SI can affect iron homeostasis in the body. However, this homeostasis requires the up-regulation or down-regulation of the absorption mechanism of iron to maintain the iron contents in serum [40]. Transferrin binds iron with high affinity, which can be measured by TIBC, and the TIBC can also reflect the amount available for binding and transferring iron in the body [41]. Furthermore, TSAT is obtained from the calculation of serum iron to the total iron-binding capacity ratio. Wang et al. found that the dietary supplementation of 5-ALA distinctly increased SI content in broilers, and that the supplementation of 10 mg/kg of 5-ALA could reduce the TIBC level [38]. The present research also showed that the supplementation of 5-ALA could significantly enhance the SI content and TSAT level in the serum of weaned rats. Yan et al. found that weaned piglets fed with 3 mg/kg 5-ALA significantly increased the SI content at d35 [25], similar to the present research. Interestingly, the present research also suggests that 50 mg/kg 5-ALA can significantly decrease the TIBC level in weaned rats, which is inconsistent with the previous study on weaned pigs [13] and cows [42]. These previous studies showed that the addition of 5-ALA either had no effects or caused an increasing trend for the TIBC level. The reasons for the inconsistency of these results may be related to factors such as the extraction method of 5-ALA, the proportion of components and the physiological state of animals, and the specific mechanism of action, which requires further study. As a result, 5-ALA has the potential to improve the iron storage in rat liver or blood by affecting the TIBC level, SI content, and TSAT level.

Hepcidin is an antibacterial peptide secreted explicitly by hepatocytes, which presents a broad spectrum of antimicrobial activity, regulates iron metabolism and maintains iron homeostasis, and controls iron absorption and distribution in vivo [43,44,45]. Hepcidin binds to ferroportin on the surface of iron-releasing cells, induces ferroportin endocytosis and its proteolysis in lysosomes, regulates cellular iron efflux, and reduces iron conversion to transferrin [46,47]. When the body is iron deficient, Hepcidin is inhibited, resulting in more iron being transported from the intestinal cells to the serum. When iron is overloaded, iron regulation results in iron uptake by duodenal cells and reduced iron release by intestinal and macrophage cells, returning iron to normal levels. Furthermore, other extrahepatic tissues or cells can also produce Hepcidin; the macrophage secretion of Hepcidin increases after bacterial infection, reducing macrophage iron release in an autocrine or paracrine manner [48]. It has been reported that Hepcidin in serum is significantly associated with iron status. Hepcidin in serum can be a valuable indicator for assessing body iron status [49]. The present study showed that weaned rats fed with 5-ALA had a significantly higher level of Hepcidin in the liver and serum compared to the control group, which suggests that 5-ALA has positive effects on Hepcidin in the body. There are also few studies on the effects of 5-ALA on the Hepcidin concentration in the serum and liver, and some studies have focused on novel iron supplements. Dong et al. found that the expression of *Hepcidin* mRNA in the liver was significantly decreased in iron-deficient piglets; in contrast, the expression of *Hepcidin* mRNA in the liver and the Hepcidin concentration in the serum were also significantly elevated in iron-injected (150 mg dextran) groups [50]. Mazgaj et al. measured the concentration of active Hepcidin-25 in piglet plasma to examine whether the hepatic expression pattern of Hepcidin reflects circulating Hepcidin peptide levels in the blood and found that the trend of Hepcidin concentration expression in plasma is similar to that in the liver. The microsomal ferric pyrophosphate (60 mg Fe/day) supplementation in sow diets can also significantly increase the Hepcidin concentration of piglets in the liver compared to the control [51]. The above novel iron supplementation is all iron with a high biological potency, which is better for absorption by the body and helps sustain Hepcidin expression. Therefore, we speculate that the high expression mechanism of Hepcidin in the animal liver or serum is similar to that of novel iron supplements. A possible mechanism is that 5-ALA is a precursor for heme biosynthesis and that the exogenous addition of 5-ALA can stimulate heme production by eliminating a process involving 5-ALA synthase; moreover, it may affect the secretion of Hepcidin by altering the iron content of the organism [24,30]. The ferrous iron may be directly converted to heme iron during this reaction, which is an excellent source of iron for more efficient absorption and utilization [13]. Nevertheless, this specific mechanism requires further detailed investigation.

Following previous discussions, it is known that Hepcidin is usually synthesized by hepatocytes and secreted into the bloodstream, which can regulate the delivery of iron into the circulation from macrophages, duodenal enterocytes, and hepatocytes [52]. Some studies have also confirmed that it can improve the iron status of sows [30], broilers [38], weaned piglets [25], cows [42]. Since iron metabolism in the body is controlled by the *Hepcidin* gene, BMP/SMADS signaling is considered to be the most crucial signaling pathway for Hepcidin activation, which involves the up-regulation or down-regulation of genes. 5-ALA may affect the expression of *Hepcidin* mRNA in the liver. Therefore, the expression of *Hepcidin* mRNA was measured to establish the impact of 5-ALA on *Hepcidin* mRNA. It is showed that the expression of the *Hepcidin* mRNA in the liver of weaned rats was up-regulated by dietary 5-ALA supplementation in the current study. Based on the mouse model, Nicolas et al. found that the transcript level of *Hepcidin* mRNA was up-regulated during iron overload and down-regulated during iron deficiency [53]. Another study also has demonstrated similar results and found that *Hepcidin* mRNA is up-regulated by iron overload and down-regulated in hypoxia/anemia [54]. Compared with piglets without iron supplementation, the *Hepcidin* mRNA expression in the liver of piglets with 150 mgFe/kg BW supplementation was higher [55]. Based on the results of the abovementioned studies, the diets supplemented with 5-ALA for pregnant rats in the present experiment may increase the iron content of weaned rats, thereby affecting the expression of the *Hepcidin* gene. The possible underlying mechanism was related to the *Hepcidin* gene regulating the release of iron ions from different cells; the *Hepcidin* gene controls iron absorption and is released from tissue storage through these pathways [56]; meanwhile, the change of iron content is fed back to the iron metabolism system in time, which affects the expression of *Hepcidin* mRNA. The transferrin receptor (TfR1) is a major player in maintaining cellular iron homeostasis [57]. The present research found that the 5-ALA diet can decrease the expression of *TfR1* mRNA in the liver of weaned rats. Iron regulatory protein (IRP) activity can indirectly affect the expression of the *Hepcidin* genes by regulating the expression of *TfR1* genes in hepatocytes. When the body’s iron is overloaded, IRPs do not bind IREs, resulting in the stability of transferrin receptor messages being decreased, decreasing the expression of *TfR1* genes [58].

## 5. Conclusions

5-ALA supplementation in the diet of female rats improved the number of RBCs, HGB concentration, and HCT level in weaned rats. The concentration of Hepcidin in the serum and liver of weaned rats was also significantly increased. The SI content and TSAT level in the weaned rats’ serum and the *Hepcidin* mRNA in the liver of weaned rats were increased by female rats fed with the 5-ALA diet. In addition, female rats fed with the 5-ALA diet reduced the level of TIBC in the serum and the level of *Tfr1* mRNA in the liver of the weaned rats. Under the conditions of this experiment, it is appropriate to add 50 mg/kg of 5-ALA to the diet of female rats. The next step will be to carry out the verification test on sows according to this experimental protocol. We speculate that 5-ALA, as a potential feed additive, will improve piglet iron content.

## Figures and Tables

**Table 1:** Composition and nutrient contents of basal diet (as feed).

Item	Content
Ingredients, %	
Corn starch	53.83
Casein	20.00
Sucrose	9.00
Soybean oil	7.00
Fiber	5.00
Mineral mixture ^1^	3.50
Vitamin mixture ^2^	1.00
L-Tryptophan	0.02
DL-Methionine	0.40
Choline bitartrate	0.25
Total	100.00
Nutrient levels	
DE/(MJ/kg)	16.46
Crude protein, %	17.96
Tryptophan, %	0.29
Methionine, %	0.90
Iron, mg/kg ^3^	277.00

^1^ Mineral premix provided the following per kg of diets: Cu 5 mg, Mn 50 mg, Zn 50 mg, I 0.15 mg, Se 0.15 mg, Ca 13 g, P 0.36 g, Mg 0.5 g, K 3.6 g, NaCl 3.6 g. ^2^ Vitamin premix provided the following per kg of diets: VA 4000 IU, VD 1000 IU, VE 80 mg, VK 1 mg, thiamine 6 mg, VB_6_ 6 mg, nicotinic acid 30 mg, folic acid 2 mg, D-calcium pantothenate 16 mg, VB_12_ 0.25 mg, biotin 0.2 mg. ^3^ Iron was the measured value in the basal diet; other nutrient levels were calculated value.

**Table 2:** Sequence of the object primers.

Genes	Primer Sequence (5′ to 3′)	Length/bp	GeneBank No.
*β-actin*	F: TCTACAATGAGCTGCGTGTG	100	NM_031144.3
	R: ACATGGCTGGGGTGTTGAA		
*Fpn1*	F: TCATTGGCTGTGGTTTCATT	228	AF394785
	R: ATTCAAGTTCACGGATGTTAGAG		
*TfR1*	F: CGAAGTCCAGTGTGGGAACA	140	NM_022712.1
	R: GGCACCAACAGCTCCATAGT		
*Hepcidin*	F: TGATGCTGAAGCGAAGGAAG	116	NM_053469.1
	R: AAGGCTCTTGGCTCTCTATGTTAT		

**Table 3:** Effects of 5-ALA on hematological indices in weaned rats.

Items ^1^	CON	5-ALA50	5-ALA100 ^2^	SEM ^3^	*p*-Value
RBC, 10^12^/L	5.40 ^b^	5.58 ^b^	5.82 ^a^	0.035	0.003
HGB, g/L	111.75 ^c^	116.75 ^b^	122.00 ^a^	0.486	<0.001
HCT, %	39.23 ^b^	41.95 ^a^	43.35 ^a^	0.445	0.013

^a–c^ Means within a row with different superscripts differ significantly (*p* < 0.05). ^1^ RBC = red blood cell; HGB = hemoglobin; HCT = hematocrit. ^2^ Dietary treatments were as follows: CON: control group, basal diet; 5-ALA50: basal diet + 50 mg/kg 5-ALA; 5-ALA100: basal diet + 100 mg/kg 5-ALA. ^3^ SEM = standard error of means for treatment effect.

**Table 4:** Effects of 5-ALA on the iron-related indicators of weaned rats.

Items ^1^	CON	5-ALA50	5-ALA100 ^2^	SEM ^3^	*p*-Value
Liver, μg/g	540.55	669.66	657.61	21.031	0.085
SI, μmol/L	28.73 ^b^	34.81 ^a^	35.81 ^a^	0.547	0.001
TIBC, μmol/L	76.33 ^a^	65.88 ^b^	75.32 ^a^	0.915	0.002
TSAT, %	37.66 ^c^	52.89 ^a^	47.72 ^a^	1.050	0.001

^a–c^ Means within a row with different superscripts differ significantly (*p* < 0.05). ^1^ SI = serum iron; TIBC = total iron-binding capacity; TSAT = transferrin saturation. ^2^ Dietary treatments were as follows: CON: control group, basal diet; 5-ALA50: basal diet + 50 mg/kg 5-ALA; 5-ALA100: basal diet + 100 mg/kg 5-ALA. ^3^ SEM = standard error of means for treatment effect.

**Table 5:** Effects of 5-ALA on the content of Hepcidin in serum and liver of weaned rats.

Items	CON	5-ALA50	5-ALA100 ^1^	SEM ^2^	*p*-Value
Serum, μg/L	105.11 ^c^	149.88 ^a^	140.78 ^b^	0.662	<0.001
Liver, μg/L	56.46 ^b^	73.68 ^a^	72.18 ^a^	0.512	<0.001

^a^^–c^ Means within a row with different superscripts differ significantly (*p* < 0.05). ^1^ Dietary treatments were as follows: CON: control group, basal diet; 5-ALA50: basal diet + 50 mg/kg 5-ALA; 5-ALA100: basal diet + 100 mg/kg 5-ALA. ^2^ SEM = standard error of means for treatment effect.

## Data Availability

Not applicable.
